# ECMO in adult patients with severe trauma: a systematic review and meta-analysis

**DOI:** 10.1186/s40001-023-01390-2

**Published:** 2023-10-10

**Authors:** Yangchun Zhang, Li Zhang, Xihua Huang, Na Ma, Pengcheng Wang, Lin Li, Xufeng Chen, Xueli Ji

**Affiliations:** Emergency Department, The First Affiliated Hospital of Nanjing Medical University, Nanjing, Jiangsu China

**Keywords:** Trauma, Extracorporeal membrane oxygenation (ECMO), Meta-analysis

## Abstract

**Background:**

Severe trauma can result in cardiorespiratory failure, and when conventional treatment is ineffective, extracorporeal membrane oxygenation (ECMO) can serve as an adjunctive therapy. However, the indications for ECMO in trauma cases are uncertain and clinical outcomes are variable. This study sought to describe the prognosis of adult trauma patients requiring ECMO, aiming to inform clinical decision-making and future research.

**Methods:**

A comprehensive search was conducted on Pubmed, Embase, Cochrane, and Scopus databases until March 13, 2023, encompassing relevant studies involving over 5 trauma patients (aged ≥ 16 years) requiring ECMO support. The primary outcome measure was survival until discharge, with secondary measures including length of stay in the ICU and hospital, ECMO duration, and complications during ECMO. Random-effects meta-analyses were conducted to analyze these outcomes. The study quality was assessed using the Joanna Briggs Institute checklist, while the certainty of evidence was evaluated using the Grading of Recommendations, Assessment, Development and Evaluations (GRADE) approach.

**Results:**

The meta-analysis comprised 36 observational studies encompassing 1822 patients. The pooled survival rate was 65.9% (95% CI 61.3–70.5%). Specifically, studies focusing on traumatic brain injury (TBI) (16 studies, 383 patients) reported a survival rate of 66.1% (95% CI 55.4–76.2%), while studies non-TBI (15 studies, 262 patients) reported a survival rate of 68.1% (95% CI 56.9–78.5%). No significant difference was observed between these two survival comparisons (p = 0.623). Notably, studies utilizing venoarterial extracorporeal membrane oxygenation (VA ECMO) (15 studies, 39.0%, 95% CI 23.3–55.6%) demonstrated significantly lower survival rates than those using venovenous extracorporeal membrane oxygenation (VV ECMO) (23 studies, 72.3%, 95% CI 63.2–80.7%, p < 0.001). The graded assessment of evidence provided a high degree of certainty regarding the pooled survival.

**Conclusions:**

ECMO is now considered beneficial for severely traumatized patients, improving prognosis and serving as a valuable tool in managing trauma-related severe cardiorespiratory failure, haemorrhagic shock, and cardiac arrest.

**Supplementary Information:**

The online version contains supplementary material available at 10.1186/s40001-023-01390-2.

## Background

Severe trauma is a significant global health issue, particularly for young adults, with high mortality rates [1]. Early post-traumatic deaths are commonly caused by cardiac arrest, haemorrhagic shock, and traumatic brain injury, while multi-organ failure, including cardiopulmonary failure and acute respiratory distress syndrome (ARDS), is often responsible for late deaths [2–4]. Extracorporeal membrane oxygenation (ECMO) provides effective support for respiratory and circulatory function by oxygenating venous blood outside the body and returning it through a pump. ECMO assumes the role of an support when conventional therapeutic interventions fall short in addressing circulatory and respiratory failure. Venovenous (VV) ECMO and venoarterial (VA) ECMO are two perfusion methods used, with VV ECMO providing respiratory support and VA ECMO providing both respiratory and circulatory support [5–7]. While ECMO use continues to expand in non-trauma scenarios, its application in trauma patients remains controversial in many centers [8]. Factors such as limited resources, anticoagulation during perfusion, haemorrhage, thrombosis, limb ischaemia, traumatic brain injury, and limited technical expertise contribute to the restricted usage of ECMO in trauma patients [9].

In recent years, the use of ECMO in trauma has increased year on year as continuous improvements in ECMO technology [10], such as the implementation of new anticoagulation strategies, have emerged as a proactive approach to reducing complications in ECMO patients [11–13]. While there are no formal guidelines, clinical consensus acknowledges the potential benefits of ECMO as a life-saving support for severely traumatized patients. However, there have been limited studies on this topic, mostly retrospective, leading to varying reports on the scope of application and survival rates [14–16]. In light of the diverse nature of ECMO’s application, resource implications, and reported outcomes in severe trauma management, we conducted a systematic review of the literature to provide guidance for clinical decisions and future research endeavors.

## Methods

This study followed the Preferred Reporting Items for Systematic Reviews and Meta-Analyses (PRISMA) statement guidelines [17] and was prospectively registered in PROSPERO (CRD 42023406004).

### Search strategy

We conducted a thorough search of the Pubmed, Embase, Cochrane, and Scopus databases until 13 March 2023. Our search utilized various medical subject terms, keywords, and their variants, such as 'Extracorporeal Membrane Oxygenation', "Extracorporeal Life Support", "ECMO Treatment", "Injuries and Wounds", and "Trauma" (Additional file 1). Relevant articles were identified through assessing both the included studies and their references.

### Selection criteria

Following the PICOS methodology, we established specific inclusion and exclusion criteria for study selection. Eligibility was limited to studies written in English or English translations. Inclusion Criteria: (1) Studies involving 5 or more trauma patients (≥ 16 years old) receiving ECMO support; (2) Both studies with control groups and those without control groups; (3) Outcome metrics including survival to hospital discharge, ICU and hospital length of stay, duration of ECMO, and complications during ECMO; (4) Study designs including both prospective and retrospective studies. Exclusion Criteria: (1) Studies involving animals or children; (2) Studies focusing on ECMO as a bridge to delayed surgery or its application to burns; (3) Case reports to avoid potential publication bias; (4) Letters, expert opinions, and commentaries; (5) Studies lacking relevant data extraction, particularly ECMO implementation details and outcomes. To avoid duplicate patient data, studies using the Extracorporeal Life Support Organisation (ELSO) registry were not included. Larger studies with overlapping patient data were included in the primary meta-analysis. Two independent reviewers (Y.Z. and N.M.) conducted the initial screening, resolving conflicts through consensus or a third-party reviewer (X.J).

### Data collection

Two independent reviewers (Y.Z. and P.W) collected data using a predetermined extraction form, resolving conflicts through consensus or a third-party reviewer (X.J). The collected data included study characteristics (design, duration, publication year, country), patient demographics (number, gender ratio, age), pre-ECMO characteristics (injury severity score [ISS], partial pressure of arterial oxygen versus fraction of inspired oxygen [PaO2/FiO2], Sequential Organ Failure Assessment [SOFA] score, mechanism of injury, presence of traumatic brain injury [TBI] and cardiac arrest [CA]), ECMO characteristics (type, initiation time, duration, anticoagulation strategy), survival (hospitalization, time of death), and relevant clinical outcomes (intensive care unit [ICU] and hospital length of stay [LOS], ECMO complications).

### Assessment of risk of bias and certainty of evidence

We utilized the Joanna Briggs Institute (JBI) list of case series and cohort studies (Additional file 2) to evaluate the quality of the included studies. Statistical heterogeneity was assessed through I2 statistics, chi-square tests, and visual examination of forest plots. The certainty of the evidence was evaluated using the Grading of Recommendations, Assessment, Development, and Evaluation (GRADE) methodology [18], with the assistance of the online GRADEpro app (https://www.gradepro.org [accessed 16 July 2023]).

### Outcomes of interest

The primary outcome assessed in this study was survival to hospital discharge, while secondary outcomes included ICU and hospital length of stay, duration of ECMO, and complications during ECMO.

### Statistical analysis

Statistical analysis of the pooled data was performed using STATA 14.0, with conversion of median, interquartile range, or extreme values to means and standard deviations [19]. A random-effects meta-analysis was conducted to account for expected heterogeneity due to diverse mechanisms and manifestations of injury, along with the lack of standardized guidelines for ECMO patient selection and management. Confidence intervals (CIs) at 95% were calculated [20, 21]. Survival outcomes were presented as combined proportions, and persistence outcomes as combined means, both with corresponding 95% CIs.

Subgroup analyses involved geographic location (Asia, Europe, and North America), type of injury (traumatic brain injury or other), and type of ECMO initiation (VV or VA), incorporating continuity correction for studies with zero events. Sensitivity analyses explored sources of heterogeneity for the primary outcome of survival to hospital discharge, and publication bias was assessed using funnel plots and Egger’s test.

## Results

### Eligible studies and study characteristics

**Fig. 1:**
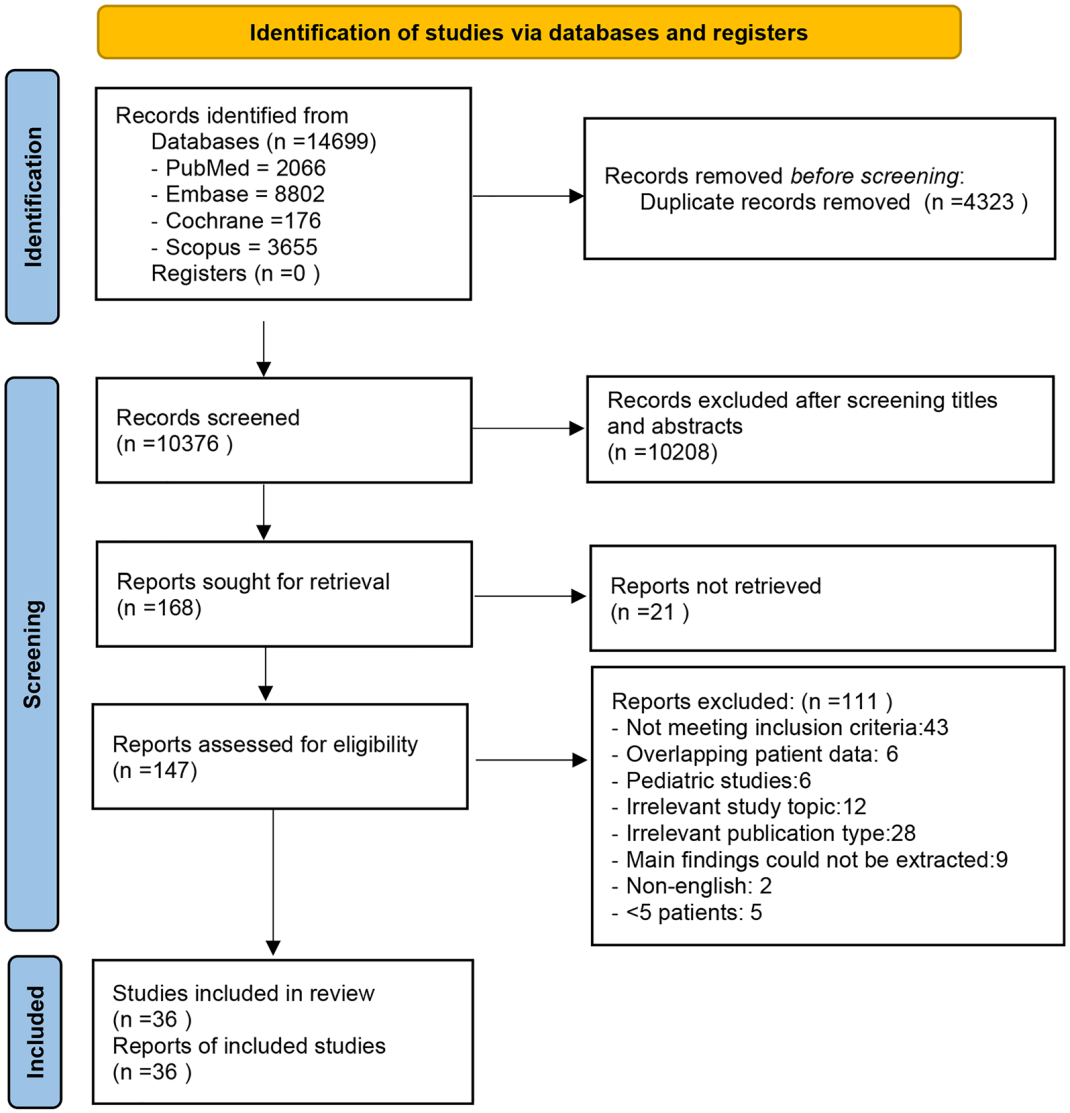
PRISMA 2020 flow diagram for the meta-analysis

A total of 14,699 records were initially identified, of which 4323 duplicate articles were removed prior to screening. An additional 10,208 studies were excluded after screening titles and abstracts. After assessing the full text, 111 more studies were removed. Eventually, a total of 36 eligible publications [3–5, 8–10, 14–16, 22–48] with 1822 patients were included in this meta-analysis. The PRISMA 2020 flow chart for this study is presented in Fig. 1.

All included studies were retrospective and observational, consisting of 2 propensity score-matched studies, 12 single-centre retrospective case series, 6 multicentre regression cohort studies, and 18 single-centre retrospective cohort studies. The combined mean age across 34 studies (1757 patients) was 35.5 years (95% CI 33.6–37.4), with a male proportion of 84.2% (95% CI 82.3–86.1%) reported in 31 studies (1428 patients). Cardiac arrest was observed in 14.4% of patients (95% CI 4.7–27.3%) in 20 studies involving 71 patients. The ISS score was reported in 29 studies comprising 1640 patients with a mean value of 34.9 (95% CI 31.7–38.1). Pre-ECMO PaO2/FiO2 was reported in 16 studies involving 333 patients, showing a value of 58.47 (95% CI 55.13–61.80). Furthermore, 10 studies including 244 patients reported a SOFA score of 10.18 (95% CI 6.87–13.49) (Table 1). The included studies presented different recorded times of ECMO onset, encompassing from injury to ECMO, admission to ECMO, emergency to ECMO, and ARDS onset to ECMO. Additional file 3 provides detailed information on these characteristics.

**Table 1:** Baseline demographics of studies included for systematic review

First author	Year of publish	Country/District	Sample Size	Age-Years*	Male patients n (%)	TBI	CA	ISS*	SOFA	P/F ratio*	Type of trauma
Mader	2023	Germany	134	49.8 ± 20.1	108 (80.6)	134	NR	35.9 ± 14.6	NR	NR	Penetrating trauma1 Road traffic74 Low fall28 High fall22
Hatfield	2023	USA	118	30 (22–46)	92 (78.0)	118	0	29 (17–41)	NR	NR	NR
Weidemann	2022	Germany	19	28 ± 11	15 (78.9)	14	0	45 ± 13	NR	70.22 ± 26.8	Car crash 8 Fall from great height 3 Motorcycle crash 5 Knife attack 1 Mechanical bruising trauma 1 Pedestrian hit by a car 1
Trivedi	2022	USA	7	32.1 ± 8.7	7 (100.0)	1	0	NR	NR	NR	Motor vehicle accident 6 Crush injury 1
Salas	2022	USA	15	30.6 [17–57]	13 (86.7)	5	0	34.1 ± 11.5	NR	53 (27–76)	Motor vehicle collision 9 Motorcycle collision 3 Fall 1 Auto-versuspedestrian accidents 2
Lee	2022	Korea	16	47.5(34.3–71.3)	13(81.3)	0	0	23.5 (10.8–29.0)	NR	60 (50–60)	Pedestrian struck by a motor vehicle 4 Motor vehicle crashes 3 Motorcycle crashes 5 Falls 2 Crushing 1
Kim	2022	Korea	21	45.0 ± 17.8	18 (85.7)	5	0	28.9 ± 11.0	NR	55.4 ± 13.1	Traffic accident 16 Fall 2 Crushing 2 Stab wound 1
Eisenga	2022	USA	10	31 [24–63]	NR(NR)	1	1	NR	NR	NR	NR
Brewer	2022	USA	12	33.6 ± 4.0	NR(NR)	NR	0	27.6 ± 6.0	NR	NR	NR
Al-Thani	2022	Qatar	22	29.6 ± 13.8	19 (86.4)	15	NR	30.6 ± 12.3	9.4 ± 4.8	NR	Motor vehicle crash 11 Pedestrian 7 Fall from height 3 Struck by a heavy Object 1
Parker	2021	USA	13	28 (25–37.5)	11 (84.6)	13	0	48 (33.5–66)	NR	58 (47–74.5)	NR
Henry	2021	USA	97	35 (22–51)	79 (81.4)	28 (-)	NR	27 (17–34)	NR	NR	NR
Lee	2020	Korea	42	41 (18.75–52.75)	37 (88.1)	5	21	NR	11.50 (4.0–19.0)	61.5 (49.7–81.7)	Car accident 9 Near drowning 18 Gunshot wound 1 Intoxication 2 Crushing injury 5 Fall down 4 Hanging 1 Stabbed wound 2
Huang	2020	USA	12	31 (27.3–38.0)	11 (91.7)	NR	3	28 (18.8–39.0)	NR	NR	Penetrating 3 Blunt 8 Mixed 1
Guttman	2020	USA	269	34.4 ± 14.8	231 (85.9)	82	NR	30.6 ± 14.6	NR	NR	Fall 25 MVC 119 Motorcycle 33 Pedestrian/cyclist 23 Other blunt 23 Firearm 38 Cut/pierce 8
Akhmerov	2020	USA	522	32.0 ± 17.4	422 (80.8)	NR	NR	26.8 ± 15.1	NR	NR	Blunt 362 Penetrating 78 Burn 38 Other 42
Kruit	2019	UK	52	34.4 ± 15.4	42 (80.8)	19	NR	35 (25–56)	12 (5–13)	62.2 (54.7–73.3)	Road traffic collision (car) 18 Road traffic collision (pedestrian) 8 Fall 8 Road traffic collision (motorbike) 7 Road traffic collision (cyclist) 3 Assault 4 Helicopter crash 1 Speedboat crash 1
Wu	2018	Taiwan	36	36 (27–49)	27 (75.0)	NR	8	29 (19–45)	NR	NR	Stabbing 1 Traffic accident 23 Electrocution 1 Falling 5 Compression injury 1
Strumwasser	2018	USA	7	38 [23–63]	7 (100.0)	NR	3	34 [16–54]	NR	55 [30–167]	Motorcycle collision 5 Gunshot wound 2
Menaker	2018	USA	18	28.5 ( 24–43)	15 (83.3)	NR	NR	27 (21–41)	NR	61 (50–70)	12 (67%) patients had a blunt mechanism of injury
Grant	2018	USA	19	NR	NR(NR)	NR	NR	NR	NR	NR	Gunshot wound 4 Assault 1 Crush 1 Traffic Accidents 6 Fall 3 Stab wound 1
Ull	2017	Germany	49	49.9 (16.6–86.2)	44 (89.8)	NR	NR	NR	12(6–19)	NR	NR
Kim	2017	Korea	9	48.0 (20.5–62.0)	8(88.9)	NR	2	NR	14.0 (10.5–15.5)	60.8 (47.3–71.8)	Car accident 4 Gunshot wound 1 Crush injury 2 Fall 2
Huh	2017	Korea	10	45.6 ± 22.3	7 (70.0)	NR	7	47.3 ± 21	7.4 ± 4.2	NR	MVA, motor vehicle acciden 4 TA, traffic Accident 2 Fall down 2 Penetrating injury 2
Burke	2017	USA	80	26.5 (19–41.5)	68 (85.0)	31	4	25 (16.5–33)	NR	NR	Penetrating trauma 9 Blunt trauma 66 Other mechanisms 5
Ahmad	2017	USA	46	NR	NR(NR)	11	NR	NR	NR	NR	NR
Chen	2016	Taiwan	7	31 (21–49)	6 (85.7)	4	NR	36 (27–57)	NR	63.4 ± 25.3	Traffic accidents were predominant 5 Followed by falls 2
Bosarge	2016	USA	15	36.0 (25.0–47.0)	15 (100.0)	2	NR	26.0 (17.0–34.0)	3.5 (3.5–3.75)	56.0 (15.5–69.0)	NR
Wu	2015	Taiwan	19	40.7 ± 18.7	17 (89.5)	5	NR	29.0 (25–34)	NR	60.0 (48–65)	MVC 17 Fall2
Tseng	2014	Taiwan	9	3 7(27–46)	8 (88.9)	0	8(3/8)	34(15.5–41)	NR	NR	Traffic accident 5 High voltage electric shock 1 Fall 2 Stab 1
Guirand	2014	USA	26	33.0 ± 11.5	20 (76.9)	NR	NR	29.0 ± 12.4	NR	49.6 ± 10.7	Blunt trauma 21
Ried	2013	Germany	26	29.3 ± 13.2	24 (92.3)	NR	NR	59.4 ± 11.2	11.8 ± 2.4	54 (48–65)	Traffic accident 21 Gunshot wound 3 Fall 1 Blunt 1
Bonacchi	2013	Italy	18	46.3 ± 17.6	12 (66.7)	0	11 (0)	53 ± 17 [18–75]	NR	NR	Motorcycle accident 15 Crash 1 Fall 2
Arlt	2010	Germany	10	34.8 [21–62]	8(80.0)	NR	1 (1)	65.44 ± 14.871	10.8 ± 1.3	NR	Traffic injury 8 Fall 1 Open chest injury 1
Huang	2009	Taiwan	9	35.1 ± 9.7	NR(NR)	NR	2 (1)	44.56 ± 4.93 [35–50]	12.1 ± 3.67	NR	Blunt traffic injuries 8 Penetrating injury 1
Cordell-Smith	2006	UK	28	27	24 (85.7)	NR	NR	18	NR	62	NR

*TBI* Traumatic Brain Injury, *CA* Cardiac Arrest, *ISS* Injury Severity Scale, *SOFA* Sequential Organ Failure Assessment, *P/F* partial pressure of arterial oxygen to fraction of inspired oxygen ratio [PaO2/FiO2], *NR* not reported

^*^Age, ISS and P/F ratio reported as mean ± SD, median (interquartile range) or median [range]

### Primary outcomes

**Fig. 2:**
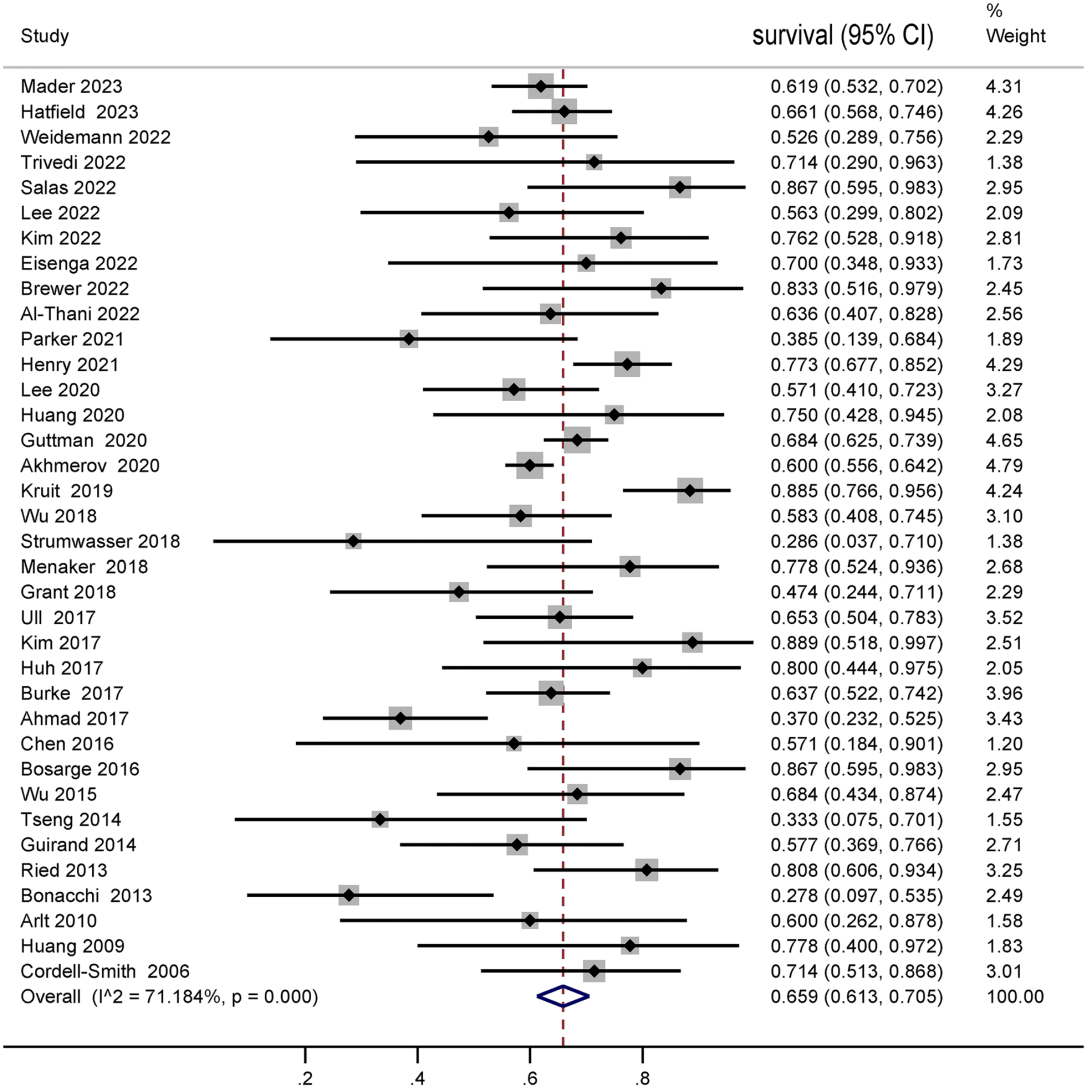
Proportion of survivors among adult patients with trauma requiring extracorporeal membrane oxygenation

The pooled survival rate before discharge in trauma patients supported with ECMO was 65.9% (95% CI 61.3–70.5%, Fig. 2), based on data from 36 studies comprising 1822 patients. Sensitivity analyses did not find any significant factors that interfered with the results, indicating stable study findings. The funnel plot showed a roughly symmetrical distribution (Additional file 5: Figure S1), and Egger’s test indicated no evidence of publication bias (*p* = 0.872).

### Subgroup analysis

The geographic region did not significantly influence outcomes in trauma patients treated with ECMO (*p* = 0.991). Survival rates were similar across North American studies (17 studies, 1286 patients), European studies (8 studies, 336 patients), and Asian studies (11 studies, 200 patients), with rates of 65.7% (95% CI 59.5–71.9%), 65.1% (95% CI 52.6–77.5%), and 66.1% (95% CI 58.0–74.2%), respectively.

Among the studies focused on traumatic brain injury (TBI) (16 studies, 383 patients), the survival rate was 66.1% (95% CI 55.4–76.2%). Similarly, studies that did not specifically focus on TBI (15 studies, 262 patients) reported a survival rate of 68.1% (95% CI 56.9–78.5%). There was no significant difference in survival rates between the two groups (*p* = 0.623).

**Fig. 3:**
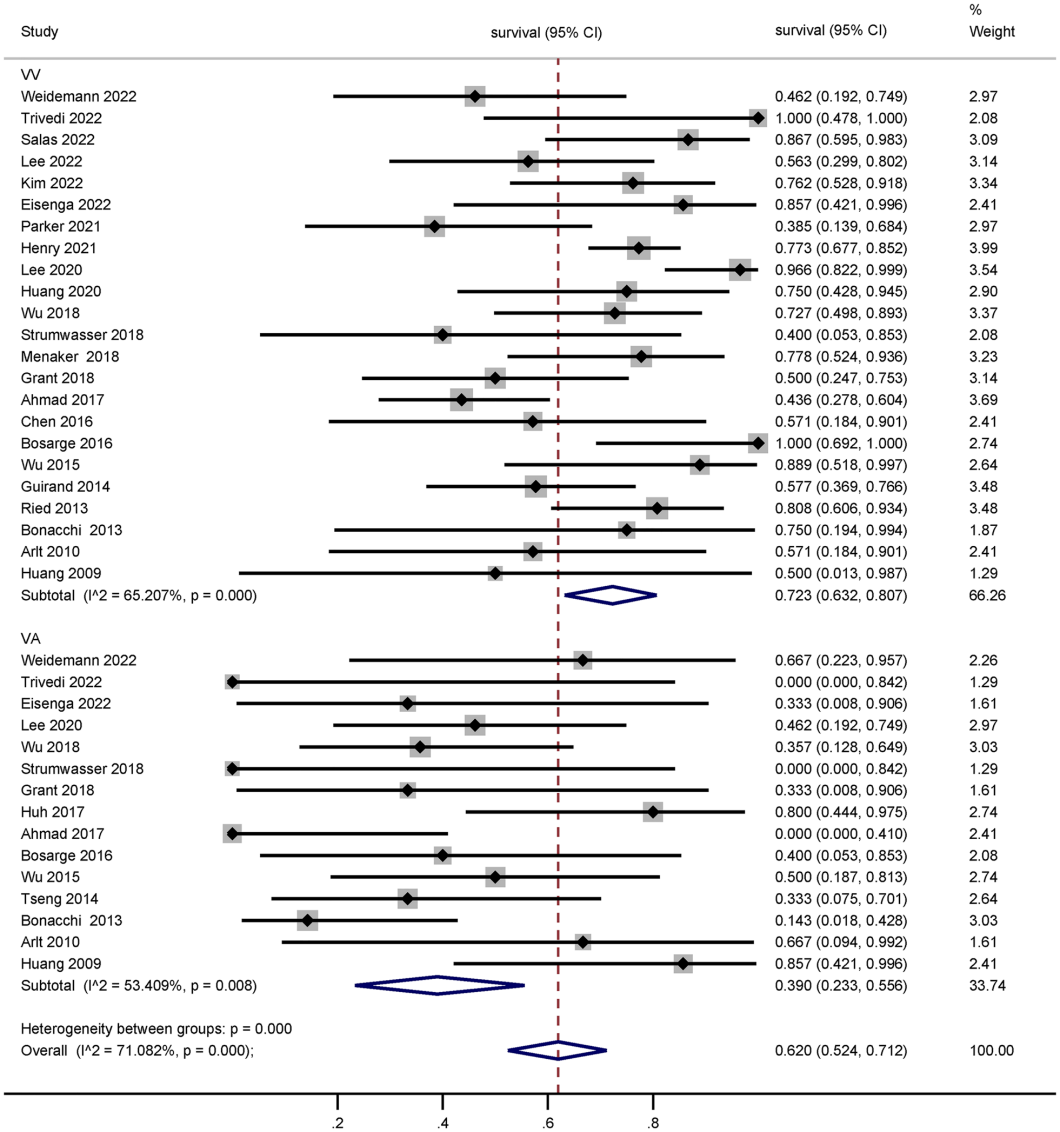
Proportion of survivors among adult patients with trauma requiring extracorporeal membrane oxygenation stratified by VA or VV

The use of VA ECMO support (15 studies) was associated with significantly lower survival rates (39.0%, 95% CI 23.3–55.6%) compared to the use of VV ECMO support (23 studies, 72.3%, 95% CI 63.2–80.7%, *p* < 0.001, Fig. 3). Detailed results of the subgroup analyses are summarized in Table 2.

**Table 2:** Result of subgroup analysis

Subgroup		Pooled survival (%)	95% CI (%)
Geographical region (*p* = 0.991)	Asia	66.1	58.0 to 74.2
	Europe	65.1	52.6 to 77.5
	North America	65.7	59.5 to 71.9
Type of trauma (*p* = 0.623)	TBI	66.1	55.4 to 76.2
	Non-TBI	68.1	56.9 to 78.5
Type of initial ECMO(*p* < 0.001)	VA	39.0	23.3 to 55.6
	VV	72.3	63.2 to 80.7

### Secondary outcomes

The pooled ICU LOS was 23.49 days (95% CI 19.90–27.08) from 19 studies with 1502 patients, and the pooled hospital LOS was 33.68 days (95% CI 29.90–37.46) from 23 studies with 1548 patients. The pooled ECMO duration was 8.17 days (95% CI 7.15–9.18) from 21 studies with 388 patients. Among the 14 studies (281 patients) reporting ECMO duration, survivors had a longer duration compared to non-survivors (3.872 days, 95% CI 1.487–6.256, *p* = 0.272). A total of 615 complications were reported in 22 studies (806 patients) treated with ECMO, with renal complications (164/806, 26.7%), infectious complications (131/806, 21.3%), and thrombotic complications (103/806, 16.8%) being the most commonly observed. Patient outcomes of the included studies are summarised in Additional file 3 and Table 3.

**Table 3:** Patient outcomes of studies included for systematic review

First author	Year of publish	Sample Size	Survivors	Survival	ICU LOS* (days)	Hospital LOS* (days)	Complications on ECMO
Mader	2023	134	83	61.9%	15 [7–29]	20[10–40]	NR
Hatfield	2023	118	78	66.1%	19.5 (8–32)	26 (11–36)	NR
Weidemann	2022	19	10	52.6%	37 ± 32	NR	Haemorrhagic 1 Cardiovascular 1
Trivedi	2022	7	5	71.4%	NR	NR	Infectious 4 Renal 3 Thrombosis 1 Other(liver failure 1)1
Salas	2022	15	13	86.7%	NR	48.9 ± 29.5	Neurologic 2 THROMBOSIS 2
Lee	2022	16	9	56.3%	23.0 (12.8–52.3)	39.5 (14.8–93.8)	0
Kim	2022	21	16	76.2%	NR	86.3 ± 50.0	0
Eisenga	2022	10	7	70.0%	NR	NR	NR
Brewer5	2022	12	10	83.3%	NR	28.4 ± 6.6	NR
Al-Thani	2022	22	14	63.6%	27.5 (2–62)	39.5 (2–81)	Renal 13 Infection 11 Thrombosis 7 Haemorrhagic 4 MOF 7 Others(thrombocytopenia 3,Mesenteric ischemia 2) 5
Parker	2021	13	5	38.5%	NR	NR	Thrombosis 4 Haemorrhagic 2
Henry	2021	97	75	77.3%	24 (14–34)	29 (16–46)	Renal 29 Neurologic 4 Infection 17 Cardiovascular 3 Limb 2 Thrombosis 17
Lee	2020	42	24	57.1%	16 (7.7–24.2)	23 (13.2–51.2)	Renal 11 Neurologic 1 Haemorrhagic 1 Limb 1 MOF 2 Other(Decubitus ulcer 2,Cholecystitis 2) 4
Huang	2020	12	9	75.0%	NR	45.5 (22–71.3)	NR
Guttman	2020	269	184	68.4%	22 (8–35)	29 (12–43)	Renal 71 Infectious 37 Neurologic 12 Cardiovascular 8 Pulmonary 88 Thrombosis 32 Other(Decubitus ulcer) 41
Akhmerov	2020	522	313	60.0%	23.1 ± 20.9	28.5 ± 26.9	NR
Kruit	2019	52	46	88.5%	NR	NR	Haemorrhagic 26 Thrombosis 21 Neurologic 3
Wu	2018	36	21	58.3%	NR	NR	Haemorrhagic 12 Neurologic 3 Renal 10
Strumwasser	2018	7	2	28.6%	NR	NR	NR
Menaker	2018	18	14	77.8%	49 (18–63)	53 (21–66)	Haemorrhagic 6 Thrombosis 10
Grant	2018	19	9	47.4%	40.5 (15.3–86)	43.5 (15.2–102)	Renal 10 Haemorrhagic 8 Thrombosis 5 Other(liver failure) 1
Ull	2017	49	32	65.3%	24(4.8–71.7)	46.6(2.9–197.6)	Mechanical 22 Haemorrhagic 7 Limb 3
Kim	2017	9	8	88.9%	22.0 (18.0–33.5)	58.0 (24.0–101.0)	NR
Huh	2017	10	8	80.0%	24.9 ± 32.7	55.5 ± 56.9	Neurologic 3 Cardiovascular 1 MOF 1
Burke	2017	80	51	63.8%	17 (5–28)	23.5 (8.5–37.5)	NR
Ahmad	2017	46	17	37.0%	NR	NR	NR
Chen	2016	7	4	57.1%	15.2 ± 7.7	26.8 ± 15.8	Renal 4 Neurologic 3 Pulmonary 1 Others (Pancreatitis)1
Bosarge	2016	15	13	86.7%	NR	43.5(30.0–93.0)	Haemorrhagic 6 Thrombosis 4
Wu	2015	19	13	68.4%	16.8 ± 9.37	NR	NR
Tseng	2014	9	3	33.3%	NR	NR	NR
Guirand	2014	26	15	57.7%	36.7 ± 7.1	39.8 ± 7.3	Renal 23 Haemorrhagic 4
Ried	2013	26	21	80.8%	17 (13–30)	24 (13–44)	Mechanical 3
Bonacchi	2013	18	5	27.8%	NR	NR	Mechanical 1 Limb 1
Arlt	2010	10	6	60.0%	NR	NR	NR
Huang	2009	9	7	77.8%	NR	43 (21–83.5)	Renal 1 Neurologic 2
Cordell-Smith	2006	28	20	71.4%	NR	NR	NR

*ECMO* Extracorporeal Membrane Oxygenation, *ICU* intensive care unit, *LOS* length of stay

^*^ICU LOS and Hospital LOS reported as mean ± SD, median (interquartile range) or median [range]

### Assessment of study quality

The quality assessment using the JBI checklist for cohort studies and case series indicated a high level of quality for the included studies in this review, with the majority scoring at least an 8 or higher (Additional file 2). Egger’s test showed non-significant publication bias. Additional file 4 provides a summary of the assessment of the level of certainty of evidence. The starting level of evidence for observational studies was high for survival outcomes. The certainty of pooled survival was high, while the certainty of ECMO duration and hospital LOS was downgraded to medium due to gross imprecision. The certainty of ICU LOS was downgraded to low due to gross inconsistency and imprecision.

## Discussion

This systematic review and meta-analysis quantitatively summarizes survival outcomes among adult trauma patients receiving ECMO therapy. Including 1822 patients from 36 studies, with a mean age of 35.5 years and a pooled survival rate of 65.9%. Previous research has shown that trauma patients receiving ECMO are typically younger and have fewer comorbidities compared to non-trauma populations. However, no significant difference in overall survival rates has been observed [20, 21, 23, 49]. Traumatic injuries can cause acute cardiopulmonary failure through direct chest trauma or indirect injuries from non-pulmonary trauma and related treatments like blood transfusions, fluid overload, and ventilator-induced acute lung injury. Managing cardiopulmonary failure in trauma patients poses unique challenges for critical care medical personnel, particularly when considering prone positioning for patients with brain injury and increased intracerebral pressure [9, 50]. Therapeutic anticoagulation during ECMO carries a risk of hemorrhage [50], which can worsen the clinical course and complicate injury patterns [28, 49], posing challenges for treatment. ECMO is not a routine life-saving intervention following trauma, but rather a salvage therapy that effectively replaces conventional treatment for young, healthy patients when conventional methods fail [3, 33, 41]. Its complexity requires a multidisciplinary healthcare team and sufficient resources for optimal implementation [26, 33, 40]. Accordingly, the ability to perform ECMO therapy has become an increasingly important quality indicator for assessing trauma centers [31]. Additionally, the aging population will bring more elderly trauma patients, presenting additional treatment challenges in the future [51].

Subgroup analysis revealed a higher survival rate of 72.3% for VV ECMO supportive therapy compared to 39.0% for VA ECMO supportive therapy. Traumatic lung injury is frequently observed in severe multiple injuries, with 10–20% of severely traumatized patients progressing to respiratory failure or ARDS with a mortality rate of 50–80% [52]. In contrast to traditional protective ventilation and prone position ventilation, VV ECMO effectively maintains gas exchange function, implements a super-protective lung ventilation strategy, prevents and reduces the adverse effects of high positive pressure and hyperventilation on lung injury, promotes lung tissue repair, and improves prognosis [53]. This is particularly beneficial for patients with severe chest trauma or those unable to undergo prone position ventilation [44, 54]. A multicenter retrospective cohort study conducted by Guirand et al. [8] compared VV ECMO and conventional mechanical ventilation (CMV) in trauma patients with acute hypoxic respiratory failure. After propensity score matching, the VV ECMO group demonstrated a significantly higher survival rate at discharge (64.7% vs. 23.5%) compared to the CMV group. However, another retrospective study investigating VV ECMO for adult ARDS treatment found no significant difference in in-hospital mortality between the VV ECMO and CMV groups after propensity score matching for baseline differences [27]. Considering factors such as the inclusion of elderly patients and lower PaO2/FiO2 ratios, among others, the investigators still recommend that critical care physicians consider VV ECMO as a salvage therapy for appropriate trauma patients [27]. The survival rate of VV ECMO in this systematic review was comparable to a previous study in 2017 [55], while the survival rate of VA ECMO was lower. This difference may be due to the inclusion of more patients with traumatic cardiac arrest (TCA). Haemorrhagic shock resulting from cardiac and macrovascular injury is the primary cause of intractable shock and cardiac arrest in trauma patients. The survival rates for TCA caused by blunt and penetrating injuries are 3.3% and 3.7% respectively, with only 1.6% of patients showing a good neurological prognosis [56]. Swol [57] conducted a review of the ELSO Registry from 1989 to 2016, focusing on ECMO support for adult trauma patients. The study found an overall survival rate of 70% and a discharge survival rate of 61%. Specifically, VV ECMO had a survival rate of 63%, VA ECMO had a survival rate of 50%, and extracorporeal cardiopulmonary resuscitation (ECPR) had a survival rate of 25%. These rates are consistent with previous ELSO registry cohorts. Notably, VA ECMO provides comprehensive hemodynamic support in refractory shock cases that do not respond to conventional therapy, effectively managing gas exchange and perfusion while physiologically stabilizing patients without the need for high-dose pressor medications [34, 45]. ECMO is crucial in reducing blood loss and preventing complications related to massive transfusion, such as fatal acidosis, hypothermia, coagulopathy triad, electrolyte abnormalities, citrate toxicity, and transfusion-associated acute lung injury [58]. Moreover, VA ECMO supports the vital signs of trauma patients, allowing for adequate time for definitive haemostatic surgery and further treatment [4]. Additionally, it may aid in preserving neurological function after cardiac arrest [34]. Although the current evidence is insufficient to support routine VA ECMO use in patients with TCA or severe shock, early initiation of VA ECMO is recommended for those with post-traumatic cardiorespiratory insufficiency, particularly younger individuals with less severe injuries (ISS < 35) and reversible tissue perfusion injury. This approach enables damage-control surgery, enhances survival rates, and improves overall prognosis [3, 4, 9, 33, 45, 46, 59]. Despite challenges such as time constraints, resource availability, high costs, and potential complications, VA ECMO presents a valuable and potentially effective emergency intervention for appropriate patients.

TBI was previously contraindicated for ECMO due to the heightened risk of intracranial hemorrhage from systemic anticoagulation [30, 60, 61]. Recently, advancements in procedures have mitigated this bleeding risk, including low-dose anticoagulation [29, 33], delayed anticoagulation (after 48–72 h) [9, 37], heparin-free application [36, 41], and improved heparin-binding circuits [21, 23]. In this study, the survival rate of TBI patients (383, 16 studies) was comparable to non-TBI patients. About 20% to 30% of TBI patients may develop ARDS [55]. Addressing the complex interplay between the brain and lungs is crucial in managing ARDS in TBI patients, given the potential negative impact of hypercapnia, hypoxia, and elevated intrathoracic pressure on the injured brain and increased intracranial pressure. Resuscitative measures for ARDS, including prone positioning, high positive end-expiratory pressure, and permissive hypercapnia, can impact intracranial pressure and lead to secondary neurological damage in TBI [14]. To prevent exacerbation of cerebral edema in trauma patients, early administration of ECMO support may be necessary specifically for severe TBI patients. ECMO offers an appealing option for TBI patients with respiratory failure as it enables the implementation of both neurological and lung-protective ventilation strategies [27]. Positive outcomes have been observed even in TBI patients undergoing craniotomy for intracranial hemorrhage [62]. Although concerns exist about possible worsening of intracranial hemorrhage with systemic anticoagulation during ECMO [60], a study conducted by Parker et al. [14] supported the use of VV ECMO therapy in TBI patients, with 6 out of 13 patients receiving systemic anticoagulation, as no deterioration in intracranial hemorrhage was observed. In a study by Kruit et al. [15], 19 TBI patients were supported on ECMO, with 12 of them receiving anticoagulation. Out of these patients, 3 deaths were unrelated to intracranial hemorrhage in the presence of ECMO anticoagulation. These findings indicate that careful implementation of ECMO supportive therapy can ameliorate secondary brain injury and improve prognosis. The decision to administer early systemic anticoagulation during ECMO in TBI patients should consider individualized factors such as the extent, stability, and location of the injury [14]. TBI alone should not be considered a contraindication for ECMO, as TBI patients receiving ECMO support tend to exhibit higher survival rates and lower rates of neurological complications. Notably, the administration of heparin anticoagulation does not escalate the risk of mortality. Moreover, advancements in ECMO systems and enhancements in circuit anticoagulation management are anticipated to foster greater utilization of ECMO as a life-saving intervention for severe TBI patients [15].

This study has several strengths, including robust inclusion and exclusion criteria, incorporating 36 studies from diverse geographical regions. Subgroup analyses were performed to explore potential sources of heterogeneity and minimize confounding. The study quality was assessed using validated tools, and the certainty of the findings was determined through grading. However, certain limitations should be acknowledged. Firstly, our review only included studies published in English, which may introduce language bias. Additionally, the variability in ECMO initiation and management across centers and regions could contribute to increased result heterogeneity. Most of the included studies were single-center retrospective studies, lacking risk adjustment or propensity score weighting, thus potentially introducing confounding factors. Nonetheless, no publication bias was detected, the majority of articles were considered high-quality based on JBI critical appraisal, and hierarchical assessments indicated a high level of certainty regarding the primary outcome. It is important to address the absence of a trial sequential analysis in our study, which could have offered valuable insights into the reliability and conclusiveness of our meta-analysis findings [63, 64]. Despite this limitation, our study provides a comprehensive analysis based on the available evidence, offering insights into the studied outcomes and their potential implications. We encourage future research to consider incorporating trial sequential analysis to enhance the robustness of findings and guide subsequent investigations.

## Conclusions

Our systematic review and meta-analysis provide substantial evidence supporting the viability of ECMO as a therapeutic approach for severely traumatized patients. It is crucial to reassess the contraindication of ECMO in managing severe cardiorespiratory failure, hemorrhagic shock, and TCA, considering its demonstrated ability to improve survival rates and overall patient prognosis, including those with traumatic brain injury TBI.

### Supplementary Information

**Additional file 1: **Search strings for respective databases.

**Additional file 2: **Joanna Briggs Institute (JBI) checklists for included studies.

**Additional file 3: **ECMO characteristics of studies included for systematic review.

**Additional file 4: **Grading of Recommendations, Assessments, Developments and Evaluations (GRADE) approach for certainty in evidence.

**Additional file 5: Figure S1.** Funnel plot for primary meta-analysis.

## Data Availability

The dataset generated and analysed during the current study can be found in the included studies and their supplementary information files.

## Abbreviations

ARDS: Acute respiratory distress syndrome
ECMO: Extracorporeal membrane oxygenation
VA: Venoarterial
VV: Venovenous
ELSO: Extracorporeal Life Support Organisation
ISS: Injury severity score
PaO2/FiO2: Partial pressure of arterial oxygen versus fraction of inspired oxygen
SOFA: Sequential organ failure assessment
TBI: Traumatic brain injury
CA: Cardiac arrest
ICU: Intensive care unit
LOS: Length of stay
JBI: Joanna Briggs Institute
GRADE: Grading of recommendations, assessment, development, and evaluations
CMV: Conventional mechanical ventilation
