# Supplementary material for: ECMO in adult patients with severe trauma: a systematic review and meta-analysis
Source: Eur J Med Res. 2023 Oct 10;28:412. doi: 10.1186/s40001-023-01390-2 (PMC10563315; doi:10.1186/s40001-023-01390-2)
Supplement: Supplementary file 1 — Additional file 1: Search strings for respective databases. [file 40001_2023_1390_MOESM1_ESM.docx]

**Additional File 1.** Search strings for respective databases

PUBMED (MEDLINE)

| 1. | "wounds injury"[Title/Abstract] OR "Trauma"[Title/Abstract] OR "Traumas"[Title/Abstract] OR "injuries wounds"[Title/Abstract] OR "research related injuries"[Title/Abstract] OR (("injurie"[All Fields] OR "injuried"[All Fields] OR "Injuries"[MeSH Subheading] OR "Injuries"[All Fields] OR "Wounds and Injuries"[MeSH Terms] OR ("Wounds"[All Fields] AND "Injuries"[All Fields]) OR "Wounds and Injuries"[All Fields] OR "injurious"[All Fields] OR "injury s"[All Fields] OR "injuryed"[All Fields] OR "injurys"[All Fields] OR "Injury"[All Fields]) AND "Research-Related"[Title/Abstract]) OR (("injurie"[All Fields] OR "injuried"[All Fields] OR "Injuries"[MeSH Subheading] OR "Injuries"[All Fields] OR "Wounds and Injuries"[MeSH Terms] OR ("Wounds"[All Fields] AND "Injuries"[All Fields]) OR "Wounds and Injuries"[All Fields] OR "injurious"[All Fields] OR "injury s"[All Fields] OR "injuryed"[All Fields] OR "injurys"[All Fields] OR "Injury"[All Fields]) AND "Research-Related"[Title/Abstract]) OR "research related injuries"[Title/Abstract] OR "research related injury"[Title/Abstract] OR "Injuries"[Title/Abstract] OR "Injury"[Title/Abstract] OR "Wounds"[Title/Abstract] OR "Wound"[Title/Abstract] OR "Wounds and Injuries"[MeSH Terms] | 1878150 |
| --- | --- | --- |
| 2. | "extracorporeal membrane oxygenations"[Title/Abstract] OR "membrane oxygenation extracorporeal"[Title/Abstract] OR "oxygenation extracorporeal membrane"[Title/Abstract] OR "ecmo treatment"[Title/Abstract] OR "ecmo treatments"[Title/Abstract] OR "treatment ecmo"[Title/Abstract] OR "ecls treatment"[Title/Abstract] OR "ecls treatments"[Title/Abstract] OR "treatment ecls"[Title/Abstract] OR "ecmo extracorporeal membrane oxygenation"[Title/Abstract] OR "extracorporeal life support"[Title/Abstract] OR "extracorporeal life supports"[Title/Abstract] OR "life support extracorporeal"[Title/Abstract] OR "venoarterial ecmo"[Title/Abstract] OR "ecmo venoarterial"[Title/Abstract] OR ("Venoarterial"[All Fields] AND "ECMOs"[Title/Abstract]) OR "venoarterial extracorporeal membrane oxygenation"[Title/Abstract] OR "venovenous ecmo"[Title/Abstract] OR "ecmo venovenous"[Title/Abstract] OR ("Venovenous"[All Fields] AND "ECMOs"[Title/Abstract]) OR "venovenous extracorporeal membrane oxygenation"[Title/Abstract] OR "Extracorporeal Membrane Oxygenation"[MeSH Terms] | 16721 |
| 3. | #1 AND #2 | 2066 |

COCHRANE

| 1. | MeSH descriptor: [Wounds and Injuries] explode all trees | 33181 |
| --- | --- | --- |
| 2. | (Wounds and Injuries):ti,ab,kw OR (Injuries and Wounds):ti,ab,kw OR (Wounds and Injury):ti,ab,kw OR (Injury and Wounds):ti,ab,kw OR (Wounds, Injury):ti,ab,kw OR (Trauma):ti,ab,kw OR (Traumas):ti,ab,kw OR (Research-Related Injuries):ti,ab,kw OR (Injuries, Wounds):ti,ab,kw OR (Injuries, Research-Related):ti,ab,kw OR (Injury, Research-Related):ti,ab,kw OR (Research Related Injuries):ti,ab,kw OR (Research-Related Injury):ti,ab,kw OR (Injuries):ti,ab,kw OR (Injury):ti,ab,kw OR (Wounds):ti,ab,kw OR (Wound):ti,ab,kw | 109755 |
| 3. | MeSH descriptor: [Extracorporeal Membrane Oxygenation] explode all trees | 293 |
| 4. | (Extracorporeal Membrane Oxygenations):ti,ab,kw OR (Membrane Oxygenation, Extracorporeal):ti,ab,kw OR (Oxygenation, Extracorporeal Membrane):ti,ab,kw OR (Oxygenation, Extracorporeal Membrane):ti,ab,kw OR (ECMO Treatment):ti,ab,kw OR (ECMO Treatments):ti,ab,kw OR (Treatment, ECMO):ti,ab,kw OR (ECLS Treatment):ti,ab,kw OR (ECLS Treatments):ti,ab,kw OR (Treatment, ECLS):ti,ab,kw OR (ECMO Extracorporeal Membrane Oxygenation):ti,ab,kw OR (Extracorporeal Life Support):ti,ab,kw OR (Extracorporeal Life Supports):ti,ab,kw OR (Life Support, Extracorporeal):ti,ab,kw OR (Venoarterial ECMO):ti,ab,kw OR (ECMO, Venoarterial):ti,ab,kw OR (Venoarterial ECMOs):ti,ab,kw OR (Venoarterial Extracorporeal Membrane Oxygenation):ti,ab,kw OR (Venovenous ECMO):ti,ab,kw OR (ECMO, Venovenous):ti,ab,kw OR (Venovenous ECMOs):ti,ab,kw OR (Venovenous Extracorporeal Membrane Oxygenation):ti,ab,kw | 1171 |
| 5. | (#1 OR #2) AND (#3 OR #4) | 176 |

EMBASE

| 1. | 'injury'/exp | 2738742 |
| --- | --- | --- |
| 2. | 'wounds and injuries':ab,ti OR 'injuries and wounds':ab,ti OR 'wounds and injury':ab,ti OR 'injury and wounds':ab,ti OR 'wounds, injury':ab,ti OR 'trauma':ab,ti OR 'traumas':ab,ti OR 'injuries, wounds':ab,ti OR 'research-related injuries':ab,ti OR 'injuries, research-related':ab,ti OR 'injury, research-related':ab,ti OR 'research related injuries':ab,ti OR 'research-related injury':ab,ti OR 'injuries':ab,ti OR 'injury':ab,ti OR 'wounds':ab,ti OR 'wound':ab,ti | 1687053 |
| 3. | #1 OR #2 | 3310566 |
| 4. | 'extracorporeal oxygenation'/exp | 39323 |
| 5. | 'extracorporeal membrane oxygenations':ab,ti OR 'membrane oxygenation, extracorporeal':ab,ti OR 'oxygenation, extracorporeal membrane':ab,ti OR 'ecmo treatment':ab,ti OR 'ecmo treatments':ab,ti OR 'treatment, ecmo':ab,ti OR 'ecls treatment':ab,ti OR 'ecls treatments':ab,ti OR 'treatment, ecls':ab,ti OR 'ecmo extracorporeal membrane oxygenation':ab,ti OR 'extracorporeal life support':ab,ti OR 'extracorporeal life supports':ab,ti OR 'life support, extracorporeal':ab,ti OR 'venoarterial ecmo':ab,ti OR 'ecmo, venoarterial':ab,ti OR 'venoarterial ecmos':ab,ti OR 'venoarterial extracorporeal membrane oxygenation':ab,ti OR 'venovenous ecmo':ab,ti OR 'ecmo, venovenous':ab,ti OR 'venovenous ecmos':ab,ti OR 'venovenous extracorporeal membrane oxygenation':ab,ti | 8284 |
| 6. | #4 OR #5 | 41049 |
| 7. | #3 AND #6 | 8802 |

SCOPUS

| 1. | TITLE-ABS-KEY ( "Wounds and Injuries" OR "Injuries and Wounds" OR "Wounds and Injury" OR "Injury and Wounds" OR "Wounds, Injury" OR "Trauma" OR "Traumas" OR "Injuries, Wounds" OR "Research-Related Injuries" OR "Injuries, Research-Related" OR "Injury, Research-Related" OR "Research Related Injuries" OR "Research-Related Injury" OR "Injuries" OR "Injury" OR "Wounds" OR "Wound" ) | 2488283 |
| --- | --- | --- |
| 2. | TITLE-ABS-KEY("Extracorporeal Membrane Oxygenations" OR "Membrane Oxygenation, Extracorporeal" OR "Oxygenation, Extracorporeal Membrane" OR "ECMO Treatment" OR "ECMO Treatments" OR "Treatment, ECMO" OR "ECLS Treatment" OR "ECLS Treatments" OR "Treatment, ECLS" OR "ECMO Extracorporeal Membrane Oxygenation" OR "Extracorporeal Life Support" OR "Extracorporeal Life Supports" OR "Life Support, Extracorporeal" OR "Venoarterial ECMO" OR "ECMO, Venoarterial" OR "Venoarterial ECMOs" OR "Venoarterial Extracorporeal Membrane Oxygenation" OR "Venovenous ECMO" OR "ECMO, Venovenous" OR "Venovenous ECMOs" OR "Venovenous Extracorporeal Membrane Oxygenation") | 23308 |
| 3. | #1 AND #2 | 3655 |
