# Supplementary material for: ECMO in adult patients with severe trauma: a systematic review and meta-analysis
Source: Eur J Med Res. 2023 Oct 10;28:412. doi: 10.1186/s40001-023-01390-2 (PMC10563315; doi:10.1186/s40001-023-01390-2)
Supplement: Supplementary file 2 — Additional file 2: Joanna Briggs Institute (JBI) checklists for included studies. [file 40001_2023_1390_MOESM2_ESM.docx]

**Additional File 2: Joanna Briggs Institute (JBI) checklists for included studies**

**JBI checklist for cohort studies**

| **Study** | **Question no.** | | | | | | | | | | | **Overall** |
| --- | --- | --- | --- | --- | --- | --- | --- | --- | --- | --- | --- | --- |
| **(1^st^ author, year)** | **1** | **2** | **3** | **4** | **5** | **6** | **7** | **8** | **9** | **10** | **11** |  |
| Mader, 2023 | 🗸 | 🗸 | 🗸 | 🗸 |  | 🗸 | 🗸 | 🗸 | 🗸 | 🗸 | 🗸 | 10/11 |
| Hatfield, 2023 | 🗸 |  | 🗸 | 🗸 | 🗸 | 🗸 | 🗸 | 🗸 | 🗸 | 🗸 | 🗸 | 10/11 |
| Lee, 2022 | 🗸 | 🗸 | 🗸 | 🗸 |  | 🗸 | 🗸 | 🗸 | 🗸 | 🗸 | 🗸 | 10/11 |
| Kim, 2022 | 🗸 | 🗸 | 🗸 |  |  | 🗸 | 🗸 | 🗸 | 🗸 | 🗸 | 🗸 | 9/11 |
| Brewer, 2022 | 🗸 | 🗸 | 🗸 |  |  | 🗸 | 🗸 | 🗸 | 🗸 | 🗸 | 🗸 | 9/11 |
| Al-Thani, 2022 | 🗸 | 🗸 | 🗸 | 🗸 | 🗸 | 🗸 | 🗸 | 🗸 | 🗸 | 🗸 | 🗸 | 11/11 |
| Henry, 2021 | 🗸 |  | 🗸 | 🗸 | 🗸 | 🗸 | 🗸 | 🗸 | 🗸 | 🗸 | 🗸 | 10/11 |
| Lee, 2020 | 🗸 | 🗸 | 🗸 | 🗸 | 🗸 | 🗸 | 🗸 | 🗸 | 🗸 | 🗸 | 🗸 | 11/11 |
| Huang, 2020 | 🗸 |  | 🗸 | 🗸 | 🗸 | 🗸 | 🗸 | 🗸 | 🗸 | 🗸 | 🗸 | 10/11 |
| Guttman, 2020 | 🗸 |  | 🗸 | 🗸 | 🗸 | 🗸 | 🗸 | 🗸 | 🗸 | 🗸 | 🗸 | 10/11 |
| Akhmerov, 2020 | 🗸 |  | 🗸 |  |  | 🗸 | 🗸 | 🗸 | 🗸 | 🗸 | 🗸 | 8/11 |
| Kruit, 2019 | 🗸 |  | 🗸 |  |  | 🗸 | 🗸 | 🗸 | 🗸 | 🗸 | 🗸 | 8/11 |
| Wu, 2018 | 🗸 | 🗸 | 🗸 | 🗸 | 🗸 | 🗸 | 🗸 | 🗸 | 🗸 | 🗸 | 🗸 | 11/11 |
| Menaker, 2018 | 🗸 |  | 🗸 |  |  | 🗸 | 🗸 | 🗸 | 🗸 | 🗸 | 🗸 | 8/11 |
| Grant, 2018 |  | 🗸 | 🗸 |  |  | 🗸 | 🗸 | 🗸 | 🗸 | 🗸 | 🗸 | 8/11 |
| Ull, 2017 | 🗸 | 🗸 | 🗸 |  |  | 🗸 | 🗸 | 🗸 | 🗸 | 🗸 | 🗸 | 9/11 |
| Kim, 2017 | 🗸 | 🗸 | 🗸 | 🗸 | 🗸 | 🗸 | 🗸 | 🗸 | 🗸 | 🗸 | 🗸 | 11/11 |
| Burke, 2017 | 🗸 |  | 🗸 | 🗸 | 🗸 | 🗸 | 🗸 | 🗸 | 🗸 | 🗸 | 🗸 | 10/11 |
| Ahmad, 2017 | 🗸 | 🗸 | 🗸 |  |  | 🗸 | 🗸 | 🗸 | 🗸 | 🗸 | 🗸 | 9/11 |
| Bosarge, 2016 | 🗸 | 🗸 | 🗸 |  |  | 🗸 | 🗸 | 🗸 | 🗸 | 🗸 | 🗸 | 9/11 |
| Wu, 2015 | 🗸 | 🗸 | 🗸 |  |  | 🗸 | 🗸 | 🗸 | 🗸 | 🗸 | 🗸 | 9/11 |
| Guirand, 2014 | 🗸 | 🗸 | 🗸 | 🗸 | 🗸 | 🗸 | 🗸 | 🗸 | 🗸 | 🗸 | 🗸 | 11/11 |
| Ried, 2013 | 🗸 | 🗸 | 🗸 |  |  | 🗸 | 🗸 | 🗸 | 🗸 | 🗸 | 🗸 | 9/11 |
| Cordell-Smith, 2006 | 🗸 |  | 🗸 |  |  | 🗸 | 🗸 | 🗸 | 🗸 | 🗸 |  | 7/11 |

**JBI checklist for case series**

| **Study** | **Question no.** | | | | | | | | | | **Overall** |
| --- | --- | --- | --- | --- | --- | --- | --- | --- | --- | --- | --- |
| **(1st author, year)** | **1** | **2** | **3** | **4** | **5** | **6** | **7** | **8** | **9** | **10** |  |
| Weidemann, 2022 | 🗸 | 🗸 | 🗸 | 🗸 | 🗸 | 🗸 | 🗸 | 🗸 | 🗸 | 🗸 | 10/10 |
| Trivedi, 2022 | 🗸 | 🗸 | 🗸 | 🗸 | 🗸 |  | 🗸 | 🗸 |  | 🗸 | 8/10 |
| Salas, 2022 | 🗸 | 🗸 | 🗸 | 🗸 | 🗸 | 🗸 | 🗸 | 🗸 | 🗸 | 🗸 | 10/10 |
| Eisenga, 2022 | 🗸 |  | 🗸 | 🗸 | 🗸 |  | 🗸 | 🗸 |  | 🗸 | 7/10 |
| Parker, 2021 | 🗸 | 🗸 | 🗸 | 🗸 | 🗸 |  | 🗸 | 🗸 |  | 🗸 | 8/10 |
| Strumwasser, 2018 | 🗸 | 🗸 | 🗸 | 🗸 | 🗸 | 🗸 | 🗸 | 🗸 | 🗸 | 🗸 | 10/10 |
| Huh, 2018 | 🗸 | 🗸 | 🗸 |  |  | 🗸 | 🗸 | 🗸 | 🗸 | 🗸 | 8/10 |
| Chen, 2016 | 🗸 | 🗸 | 🗸 | 🗸 | 🗸 | 🗸 | 🗸 | 🗸 | 🗸 | 🗸 | 10/10 |
| Tseng, 2014 | 🗸 | 🗸 | 🗸 | 🗸 | 🗸 | 🗸 | 🗸 | 🗸 | 🗸 | 🗸 | 10/10 |
| Bonacchi, 2013 | 🗸 | 🗸 | 🗸 | 🗸 | 🗸 | 🗸 | 🗸 | 🗸 | 🗸 | 🗸 | 10/10 |
| Arlt, 2010 | 🗸 | 🗸 | 🗸 | 🗸 | 🗸 | 🗸 | 🗸 | 🗸 | 🗸 | 🗸 | 10/10 |
| Huang, 2009 | 🗸 | 🗸 | 🗸 | 🗸 | 🗸 | 🗸 | 🗸 | 🗸 | 🗸 | 🗸 | 10/10 |
