# Supplementary material for: ECMO in adult patients with severe trauma: a systematic review and meta-analysis
Source: Eur J Med Res. 2023 Oct 10;28:412. doi: 10.1186/s40001-023-01390-2 (PMC10563315; doi:10.1186/s40001-023-01390-2)
Supplement: Supplementary file 3 — Additional file 3: ECMO characteristics of studies included for systematic review. [file 40001_2023_1390_MOESM3_ESM.docx]

**Additional File 3.**  ECMO characteristics of studies included for systematic review

| **First author** | **Year of publish** | **Sample Size** | **Type of initial ECMO** | **ECMO Initiated*** | **uration of ECMO* ( Days)** | **Anticoagulation** |
| --- | --- | --- | --- | --- | --- | --- |
| Mader | 2023 | 134 | NR | NR | NR | NR |
| Hatfield | 2023 | 118 | NR | Within 2 days of admission 64 (54.3%) | NR | NR |
| Weidemann | 2022 | 19 | VV 13 VA 6 | After Injury  44±75h | 11.4±9.9 | Heparin-induced anticoagulation 13 AT-III based coagulation control 5 Non-anticoagulation 1 |
| Trivedi | 2022 | 7 | VV 5 VA 2 | After Injury 9[0-22]d | 13.2±6.5 | Non-anticoagulated 1 Heparin 2 Argatroban 2 Discontinued anticoagulation 3 |
| Salas | 2022 | 15 | VV | After intubation 3.1±2.9d | 15.2±13.2 | Heparin 12 Bivalirudin 3 |
| Lee | 2022 | 16 | VV | After ARDS 2.3±0.9d(0~5) | 9.8±5.5 | For trauma patients with coagulation disorders or high risk of bleeding, the starting dose of Nafamostat mesylate is 20 mg/h, adjusted from 10 mg/h to 30 mg/h depending on the patient's condition |
| Kim | 2022 | 21 | VV | After Injury 3.5±3.5d | 9.4±5.3 | Heparin dose was adjusted to a target ACT of 150-200s For patients at risk of significant bleeding, anticoagulation was withheld until the risk of bleeding was reduced and a high flow rate of ECMO was maintained Platelet count maintained at >80,000 |
| Eisenga | 2022 | 10 | VA 3 VV 7 | After admission to hospital 6.8±8.0d | 7.5±5.0 | NR |
| Brewer | 2022 | 12 | VA 1 VV 11 | NR | 9.4±2.4 | All patients were anticoagulated with heparin at the start with at least 5000 units or titrated to an activated clotting time (ACT) of >150 seconds |
| Al-Thani | 2022 | 22 | VA 1 VV 21 | After admission to hospital 2(1.0-14)d | 9.5(1-29) | In the case of traumatic brain injury (TBI), heparin is not given 48-72 hours after the trauma |
| Parker | 2021 | 13 | NR | After Injury 5(0.75-13)d | 8(2,16) | Six patients (46%) received systemic A/C at the time of ECMO treatment |
| Henry | 2021 | 97 | VV | NR | NR | 44% received regular heparin, 51% received low molecular weight heparin and 6% received another form of anticoagulation Anticoagulation started on average 3.9±4.6 days after starting ECMO |
| Lee | 2020 | 42 | VV 29 VA 13 | After admission to hospital 3.5 (2–81)h | 6.5(3.8–9.7) | Heparin 11 (26.2) Nafamostat mesylate 31 (73.8) |
| Huang | 2020 | 12 | VV | After admission to hospital5(0.25-10)d | NR | NR |
| Guttman | 2020 | 269 | NR | NR | NR | NR |
| Akhmerov | 2020 | 522 | NR | 7.4±18.2d | NR | NR |
| Kruit | 2019 | 52 | VV 50 VA 2 | After Injury 6 (3–11 [1–24])d | NR | Anticoagulation was started in 43 patients (84%); for 36 patients (65%) this was in the first 72 hours after ECMO initiation |
| Wu | 2018 | 36 | VA 14 VV 22 | After admission to the emergency room 25(10-121)h | NR | Heparin titration strategy including pre-intubation push dose (5000 units) and continuous intravenous maintenance dose of heparin to maintain extended aPTT (45-55 seconds) during the ECMO period |
| Strumwasser | 2018 | 7 | NR | 3[1-29]d | 3[1-21] | With normal heparin, an ACT target of 150-180 s or a PTT of 65-90 s |
| Menaker | 2018 | 18 | VV | After Injury 4(0-6)d | NR | 14 (78%) patients received low-dose anticoagulation during ECMO (PTT 45-55) 4 patients did not receive any anticoagulation during ECMO |
| Grant | 2018 | 19 | NR | 5(2-13)d | 11(5-21) | 42% of patients did not receive anticoagulation |
| Ull | 2017 | 49 | VV 34 VA 4 | After admission to hospital 3.2(0-28)d | 9.1(0.1-21.4) | For traumatic brain injury with or without bleeding on cranial CT, do not give heparin for at least 48-72 hours after the traumatic event |
| Kim | 2017 | 9 | VV | 50.0(4.5-131.0)h | 6.0(5.0-7.8) | For patients at high risk of bleeding or undergoing surgery, anticoagulation is usually started after 24 hours |
| Huh | 2017 | 10 | VA | NR | NR | Considering the risk of bleeding, the dose of heparin was adjusted to a target ACT of between 150 and 200 seconds |
| Burke | 2017 | 80 | NR | After admission to hospital 45(10-212)h | NR | NR |
| Ahmad | 2017 | 46 | NR | NR | NR | Patients anticoagulated with heparin to achieve an activated clotting time between 160 and 180 seconds and/or a partial thromboplastin time of 60 to 80 seconds or 45 to 55 seconds |
| Chen | 2016 | 7 | NR | NR | NR | No heparin for at least 4 days |
| Bosarge | 2016 | 15 | NR | After admission to hospital5(2-9)d | NR | Maintenance of intravenous heparin |
| Wu | 2015 | 19 | VA 10 VV 9 | NR | 7(4-10) | 16 (84.2%) of patients maintained an activated clotting time between 180 and 200 s In patients with coagulopathy and bleeding, a heparin-free strategy allowed an activated clotting time in the range of 140 to 160 s |
| Tseng | 2014 | 9 | VA | After admission to hospital 6(4-47.5)h | 3.8(1.8-7.8) | For patients at high risk of bleeding, a "heparin minimisation" strategy (no heparin loading or maintenance dose) will be used. The duration of the "no heparin" strategy will not exceed 48 hours and high flow ECLS (blood flow > 2.5 L/min) must be maintained to reduce the risk of thrombosis |
| Guirand | 2014 | 26 | VV | After Injury4.6±5.8d | 9.3±9.5 | Continuous infusion of normal heparin to maintain an activated clotting time of 180 to 220 seconds to achieve complete anticoagulation |
| Ried | 2013 | 26 | VV | After Injury 4.5 ± 7.3 (0 to 34)d | 6.3±3.1 (<1- 13) | Avoid heparin for a certain period of time (≤48 hours) in patients at high risk of further bleeding complications or with evidence of associated intracranial haemorrhage (Glasgow Coma Score <9 and/or pathology computed tomography scan) |
| Bonacchi | 2013 | 18 | NR | After Injury 359.176±216.606（145-950）m | NR | Heparin-free ECLS was initially performed until the bleeding stopped and the patient's coagulation status was normalised |
| Arlt | 2010 | 10 | VV 7 VA 3 | NR | 5.3±3.4 | Bleeding and shock symptoms effectively treated with low dose heparin (5000 units IV) to achieve PTT twice the normal range or ACT over 150 s Heparin-free is started in those with bleeding |
| Huang | 2009 | 9 | VV 7 VA 2 | After Injury 33[4-384]h | 6.0(2.9-19) | Heparin was prescribed at 10-15 units/(kg h) and titrated to an activated clotting time (ACT) of 180-250 seconds and an activated partial thromboplastin time (aPTT) of 55-60 seconds |
| Cordell-Smith | 2006 | 28 | NR | 69h | 5.9 | Limited anticoagulation with intravenous heparin to maintain an activated clotting time of between 180 and 220 seconds |

ECMO, Extracorporeal Membrane Oxygenation; VV, Venovenous; VA, Venoarterial; NR, not reported

*ECMO Initiated and Duration of ECMO reported as mean ± SD, median (interquartile range) or median [range]
