# Supplementary material for: ECMO in adult patients with severe trauma: a systematic review and meta-analysis
Source: Eur J Med Res. 2023 Oct 10;28:412. doi: 10.1186/s40001-023-01390-2 (PMC10563315; doi:10.1186/s40001-023-01390-2)
Supplement: Supplementary file 4 — Additional file 4: Grading of Recommendations, Assessments, Developments and Evaluations (GRADE) approach for certainty in evidence. [file 40001_2023_1390_MOESM4_ESM.docx]

**Additional File 4:** Grading of Recommendations, Assessments, Developments and Evaluations (GRADE) approach for certainty in evidence

| **№ of studies** | **Certainty assessment** | | | | | | **Effect** | | | **Certainty** | **Importance** |
| --- | --- | --- | --- | --- | --- | --- | --- | --- | --- | --- | --- |
|  | **Study design** | **Risk of bias** | **Inconsistency** | **Indirectness** | **Imprecision** | **Other considerations** | **№ of events** | **№ of individuals** | **Rate (95% CI)** |  |  |
| Pooled in-hospital Survival for all ECMO patients | | | | | | | | | | | |
| 36 | observational studies | not serious | not serious^a^ | not serious | not serious | none | 1170 | 1822 | event rate 65.9 per 100 (61.3 to 70.5) | ⨁⨁⨁⨁ High | CRITICAL |
| Intensive Care Unit Length of Stay (days) | | | | | | | | | | | |
| 19 | observational studies | not serious | serious^b^ | not serious | serious^c^ | none | - | 1502 | mean 24.49 (19.9 to 27.08) | ⨁⨁◯◯ Low | CRITICAL |
| Hospital length of stay (days) | | | | | | | | | | | |
| 23 | observational studies | not serious | not serious^d^ | not serious | serious^c^ | none | - | 1548 | mean 33.68 (29.9 to 37.46) | ⨁⨁⨁◯ Moderate | CRITICAL |
| Duration of mechanical ventilation before ECMO (days) | | | | | | | | | | | |
| 21 | observational studies | not serious | not serious^d^ | not serious | serious^e^ | none | - | 388 | mean 8.17 (7.15 to 9.18) | ⨁⨁⨁◯ Moderate | IMPORTANT |

#### Explanations

a. There was some heterogeneity (I2 = 61.45%) in the point estimates. Nonetheless, the 95% CIs for the individual studies mostly overlapped with each other. Furthermore, subgroup analysis found significant differences among patient groups.

b. There was important heterogeneity. Overall, the point estimates are sparsely distributed and the 95% CI only occasionally overlap.

c. The width of the 95% CI is wide, and holds important clinical and economic implications for patients. The decision to initiate ECMO might potentially change at both ends of the 95% CI. As such, we rated down for imprecision

d. There was significant heterogeneity, and some variability in the point estimates in the forest plots. Nonetheless, most of the 95% CIs overlapped with each other.

e. The number of patients is small, below the optimal information size
