# Supplementary figures and images for: ECMO in adult patients with severe trauma: a systematic review and meta-analysis
Source: Eur J Med Res. 2023 Oct 10;28:412. doi: 10.1186/s40001-023-01390-2 (PMC10563315; doi:10.1186/s40001-023-01390-2)

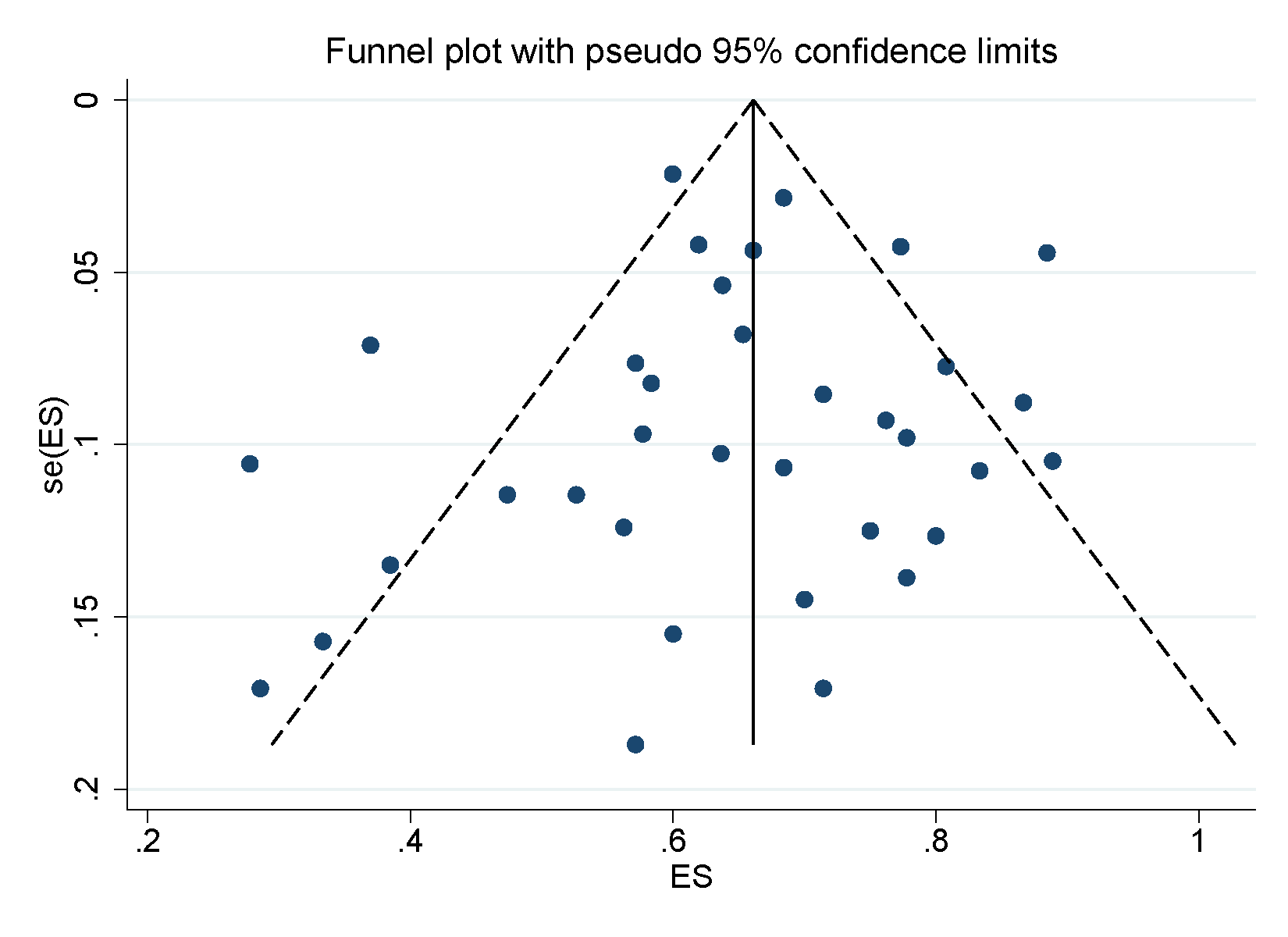

Supplement: Supplementary file 5 — Additional file 5: Figure S1. Funnel plot for primary meta-analysis. [file 40001_2023_1390_MOESM5_ESM.tif]
